# Less Carcinogenic Chlorinated Estrogens Applicable to Hormone Replacement Therapy

**DOI:** 10.3390/ijms22137222

**Published:** 2021-07-05

**Authors:** Yoshinori Okamoto, Hideto Jinno, Shinji Itoh, Shinya Shibutani

**Affiliations:** 1 Faculty of Pharmacy, Meijo University, Nagoya 468-8503, Japan; okmt@ccalumni.meijo-u.ac.jp (Y.O.); jinno@meijo-u.ac.jp (H.J.); 2 Faculty of Pharmaceutical Sciences, Hokkaido University of Science, Sapporo 006-8585, Japan; itoh-m33@hus.ac.jp; 3 Department of Pharmacological Sciences, State University of New York at Stony Brook, Stony Brook, NY 11794-8651, USA

**Keywords:** estrogen, chlorination, mammary tumor, uterotrophic activity, hormone replacement therapy, DNA damage

## Abstract

Human estrogens prescribed for hormone replacement therapy (HRT) are known to be potent carcinogens. To find safer estrogens, several chlorinated estrogens were synthesized and their carcinogenic potential were determined. A pellet containing either 2-chloro-17β-estradiol (2-ClE_2_) or 4-chloro-17β-estradiol (4-ClE_2_) was implanted subcutaneously for 52 weeks into August Copenhagen Irish (ACI) rats, a preferred animal model for human breast cancer. 17β-Estradiol (E_2_) frequently induced mammary tumors while both 2-ClE_2_ and 4-ClE_2_ did not. Their 17α-ethinyl forms, thought to be orally active estrogens, were also synthesized. Neither 2-chloro-17α-ethinylestradiol (2-ClEE_2_) nor 4-chloro-17α-ethinylestradiol (4-ClEE_2_) induced tumors. The less carcinogenic effects were supported by histological examination of mammary glands of ACI rats treated with the chlorinated estrogens. A chlorine atom positioned at the 2- or 4-position of E_2_ may prevent the metabolic activation, resulting in reducing the carcinogenicity. 2-ClE_2_ and 4-ClE_2_ administered subcutaneously and 2-ClEE_2_ and 4-ClEE_2_ given orally to ovariectomized rats all showed uterotrophic potency, albeit slightly weaker than that of E_2_. Our results indicate that less carcinogenic chlorinated estrogens retaining estrogenic potential could be safer alternatives to the carcinogenic estrogens now in use for HRT.

## 1. Introduction

Human estrogens are used in hormone replacement therapy (HRT) to alleviate menopausal symptoms and protect against osteoporosis [1,2]. Unfortunately, long-term HRT increases the incidence of breast [3,4,5,6] and endometrial cancers [7]. The risk of these cancers is correlated with the duration of HRT [4,5,6,8]. The mechanism underlying estrogen-induced carcinogenesis has not been fully explored, but both proliferative effects mediated through the estrogen receptor and/or DNA damage induced by human estrogen metabolites are significant factors in the process [9,10,11].

**Figure 1:**
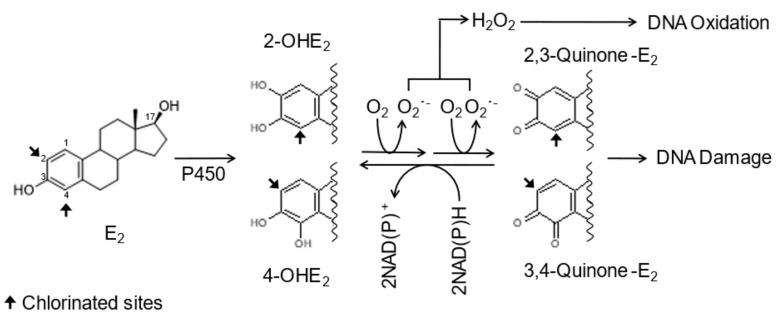
Proposed oxidative mechanism of chlorinated estrogens.

DNA damage has been detected in the tissues of rodents treated with human estrogens [12,13,14,15]. Human estrogens are primarily metabolized by cytochrome P450 enzymes to form 2- and 4-hydroxyestrogens (2- and 4-OHE) [16] (Figure 1). The catecholestrogens are oxidized further to 2,3-quinone and/or 3,4-quinone by P450 enzymes or peroxidase [16,17,18]. The 2,3-quinone of 2-OHE reacts with DNA to form 2-OHE-*N*^2^-dG and 2-OHE-*N*^6^-dA [19], which are potentially mutagenic [20]. The 3,4-quinone of 4-OHE reacts with dA and dG to form 4-OHE-1(α,β)-*N*^3^-dA and 4-OHE-1(α,β)-*N*^7^-dG, which are readily depurinated [19,21]. The resulting apurinic sites are mutagenic lesions [22], contributing to the initiation of cancer. During redox cycling, 2,3-quinone and/or 3,4-quinone are reduced back to their catecholestrogens. In the reduction reactions, free radicals produced cause oxidative DNA damage such as 8-oxo-7,8-dihydro-2′-deoxyguanosine [23], which has been detected in mammary DNA obtained from breast cancer patients [24]. Thus, DNA damages induced by human estrogens are capable of initiating breast and endometrial cancer.

**Figure 2:**
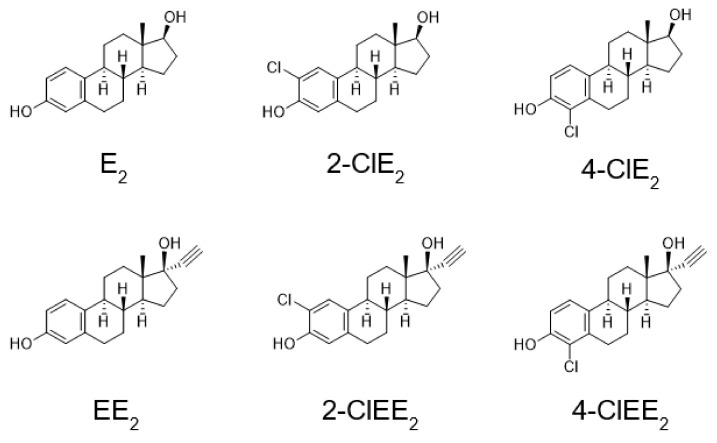
Structures of E_2_ and EE_2_ and their chlorinated compounds.

The August Copenhagen Irish (ACI) rat strain has been used as a preferred animal model for studying human sporadic breast cancer. ACI rats have a very low incidence (11% over 3 years) of spontaneous mammary tumors [25,26], which is advantageous for accurately evaluating their tumorigenicity. Indeed, E_2_ (the structure in Figure 2) [25,26,27] and 17α-ethinylestradiol (EE_2_) [28] were demonstrated to induce mammary tumors. Therefore, women receiving human estrogens for HRT may have a higher risk of developing breast and reproductive cancers.

In the present study, to prevent the metabolic activation of estrogens, a hydrogen atom at the 2- or 4-position of human estrogen was replaced by a chlorine atom. The tumorigenic and estrogenic potentials of the synthesized chlorinated estrogens (Figure 2), 2-chloro-17β-estradiol (2-ClE_2_) and 4-chloro-17β-estradiol (4-ClE_2_), were determined using rat models. Because E_2_ is inactivated when taken orally, the 17α-ethinyl formula is used for oral treatment [29,30]. The chlorinated 17α-ethinyl compounds, 2-chloro-17α-ethinylestradiol (2-ClEE_2_) and 4-chloro-17α-ethinylestradiol (4-ClEE_2_), were also synthesized and subjected to measure their tumorigenic and estrogenic potentials.

## 2. Results

### 2.1. Evidence of Mammary Tumors

**Figure 3:**
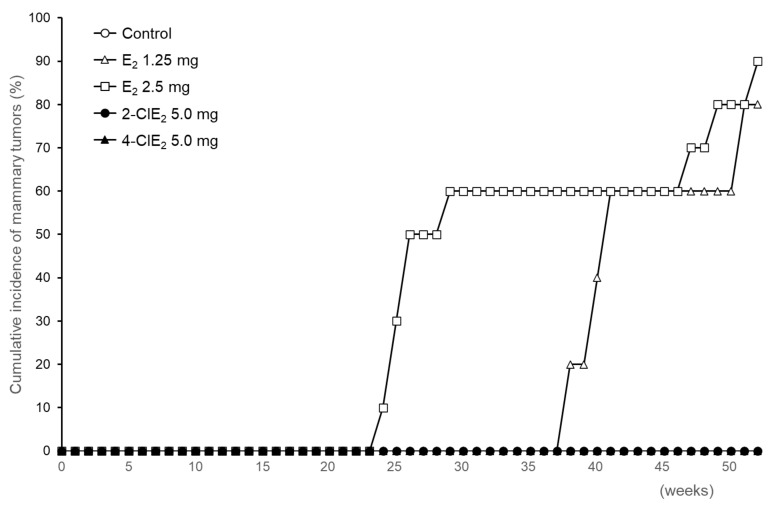
Cumulative incidence of mammary tumors in chlorinated estrogen-treated rats. Development of mammary tumors in ACI rats implanted with placebo (*n* = 5), E_2_ [1.25 mg (*n* = 5), 2.5 mg (*n* = 10)], 2-ClE_2_ [5.0 mg (*n* = 6)], or 4-ClE_2_ [5.0 mg (*n* = 6)] pellets was monitored once a week for 52 weeks.

Development of mammary tumors in ACI rats implanted with a pellet containing E_2_ or a chlorinated estrogen was monitored by palpation once a week for 52 weeks. In rats administered E_2_ (1.25 mg), palpable mammary tumors appeared around 38 weeks after pellet implantation; the cumulative incidence of the tumors was 80% (4/5) after 52 weeks (Figure 3 and Table 1). With 2.5 mg E_2_, mammary tumors appeared earlier, at 24 weeks, although the cumulative tumor incidence was 90% (9/10)—i.e., almost same as that observed with 1.25 mg E_2_. When 5.0 mg E_2_ was implanted, severe loss of body weight occurred within several weeks; therefore, the experiment with 5.0 mg E_2_ was terminated. Mammary tumors were confirmed by pathological examination as performed previously [27,31]. The body weight of rats treated with the following chlorinated estrogen increased as observed with the untreated rats. When dissected ACI rats treated with chlorinated estrogens at the end of experiment, no significant abnormality was observed in other organs including ovary and uterus. Among the 6 rats treated with 2.5 or 5.0 mg 2-ClE_2_ or 4-ClE_2_, no palpable mammary tumors were observed even after 52 weeks, as shown in the untreated rats (Figure 3 and Table 1). The 17α-ethinyl compounds, 2-ClEE_2_ and 4-ClEE_2_, also did not induce the tumors (Table 1). The data obtained by the 17α-ethinyl formula may support and strengthen the non-tumor evidence of 2-ClE_2_ and 4-ClE_2_.

### 2.2. Histological Examination of Mammary Glands

**Figure 4:**
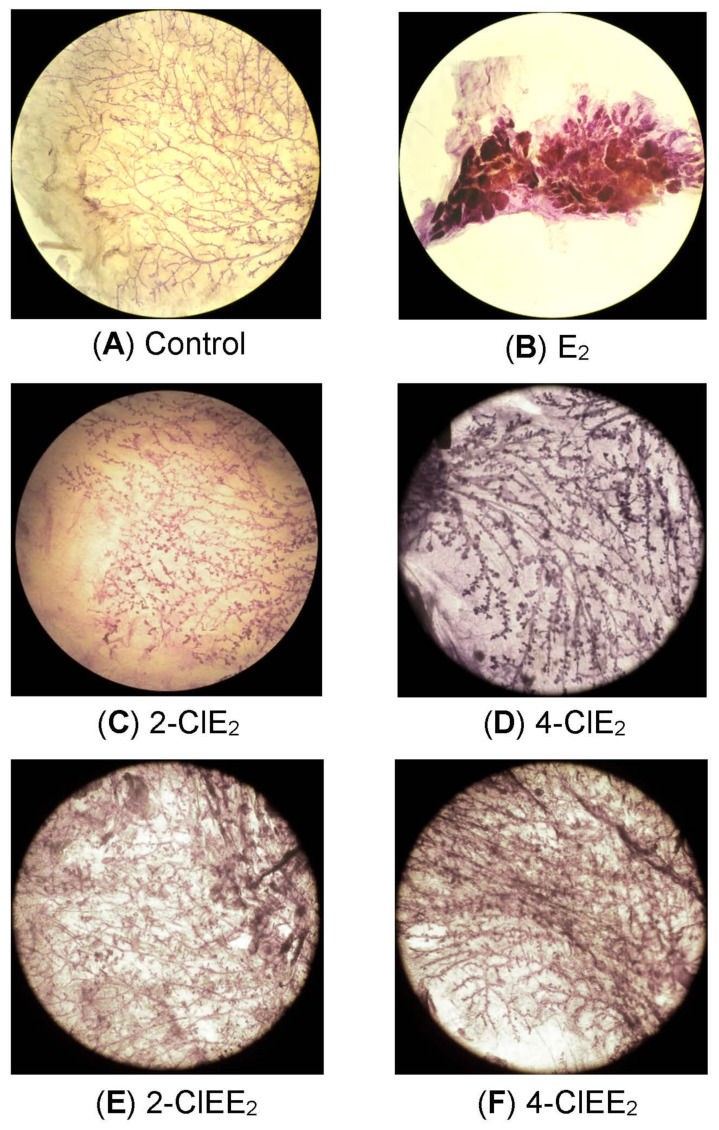
Morphological examination of mammary glands of chlorinated estrogen-treated rats. Mammary glands were collected from ACI rats at the end of the experiments. Mammary tissue of ACI rats implanted with placebo (**A**), E_2_ (2.5 mg) (**B**), 2-ClE_2_ (5.0 mg) (**C**), 4-ClE_2_ (5.0 mg) (**D**), 2-ClEE_2_ (5.0 mg) (**E**), or 4-ClEE_2_ (5.0 mg) (**F**) and stained with hematoxylin (magnification 10×).

Mammary whole-mounts of chlorinated estrogen-treated ACI rats were subjected to histological examination. With E_2_ (Figure 4B), extension of the mammary glands, branching of the ducts, and the number of end buds and alveoli were all increased. Premalignant lesions such as acinar hyperplasia was also observed in whole-mount preparations. In contrast, enlargement of the mammary glands in rats treated with 2-ClE_2_ (Figure 4C) or 4-ClE_2_ (Figure 4D) was much less than that produced by E_2_. Mammary glands of rats treated with the 17α-ethinyl forms, 2-ClEE_2_ (Figure 4E) and 4-ClEE_2_ (Figure 4F), showed similar histological results as those treated with 2-ClE_2_ and 4-ClE_2_, respectively. No precancerous lesions were detected in rats treated with any chlorinated estrogens. Neither enlargement of the mammary glands nor premalignant lesions were detected in the vehicle-treated control ACI rats (Figure 4A).

### 2.3. Uterotrophic Activity of Chlorinated Estrogens

**Figure 5:**
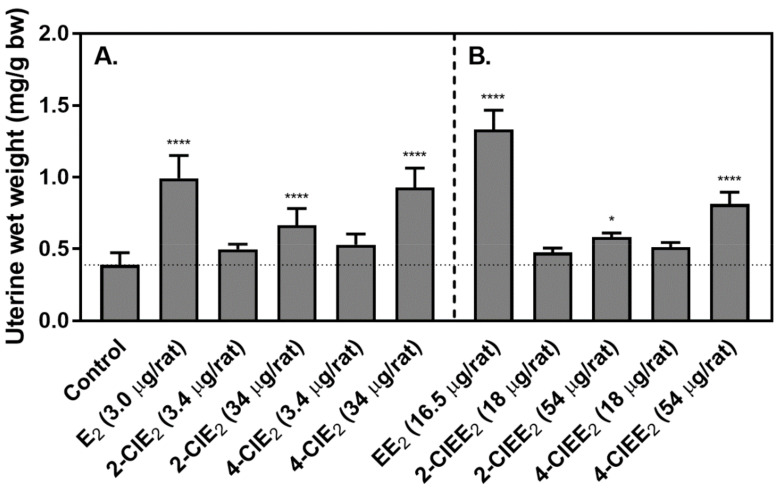
Uterotrophic potential of chlorinated estrogens on OVX-rats. (**A**) OVX-rats (4 rats/group) were treated subcutaneously for 3 days with 2-ClE_2_ (3.4 or 34 μg/rat/day) or 4-ClE_2_ (3.4 or 34 μg/rat/day). The rats treated with E_2_ (3.0 μg/rat/day) were used as positive controls. The negative control rats received vehicle only. (**B**) OVX-rats (4 rats/group) were treated orally for 3 days with 2-ClEE_2_ (18 or 54 μg/rat/day) or 4-ClE_2_ (18 or 54 μg/rat/day). The rats treated with EE_2_ (16.5 μg/rat/day) were used as positive controls. On day 4, uterine wet-weight/bw ratios were calculated and compared to that obtained for OVX-rats that received vehicle, as described in Materials and Methods. Statistical analysis (one-way ANOVA with Tukey’s post hoc test) was performed for multiple comparisons to evaluate differences; *, *p* < 0.05 (control vs. 2-ClEE_2_ (54 μg/rat)); ****, *p* < 0.0001 (control vs. E_2_ (3.0 μg/rat), 2-ClE_2_ (34 μg/rat), 4-ClE_2_ (34 μg/rat), EE_2_ (16.5 μg/rat), or 4-ClE_2_ (54 μg/rat)).

To determine the estrogenic potential of chlorinated estrogens, 2-ClE_2_ or 4-ClE_2_ was administered subcutaneously for 3 days to OVX-rats (Figure 5A). E_2_ (3.0 μg), as a positive control, increased the uterine length and thickness. With 2-ClE_2_ or 4-ClE_2_ at a dose molar equivalent of E_2_ (3.0 μg), no increase in uterine size was observed. With a 10-times molar dose (34 μg), both 2-ClE_2_ and 4-ClE_2_ promoted uterine weight gain. The uterine weight of OVX-rats treated with E_2_ was 0.99 mg/g bw and that of untreated OVX-rats was 0.39 mg/g bw. Both 2-ClE_2_ (0.67 mg/g bw) and 4-ClE_2_ (0.93 mg/g bw) showed significant uterotrophic activity.

Since 17α-ethinyl formula is designed for oral treatment, the uterotrophic potency of 2-ClEE_2_ or 4-ClEE_2_ was determined after given orally for 3 days (Figure 5B). At the dose molar equivalent to EE_2_ (16.5 μg), neither 2-ClEE_2_ nor 4-ClEE_2_ produced an increase in uterine weight. At the 3-times molar dose (54 μg), a significant increase of uterine weight was measured. The uterine weight of OVX-rats treated with EE_2_ was 1.33 mg/g bw. Both 2-ClEE_2_ (0.58 mg/g bw) and 4-ClEE_2_ (0.81 mg/g bw) showed significant uterotrophic activity.

## 3. Discussion

Trace amounts of chlorinated estrogens are produced in the environment as byproducts of reactions between estrogens and hypochlorous acid in sewage treatment plants [32,33]; their estrogenic potency was detected using an in vitro yeast two-hybrid assay with the estrogen receptor α. However, the biological properties of chlorinated estrogens in vivo are poorly understood. In the present study, the carcinogenic potential of chlorinated estrogens was determined using estrogen-sensitive ACI rats implanted with their pellets for one year. As reported previously [25,27,31], E_2_ (1.25 mg or 2.5 mg) was a potent inducer of mammary tumors (Figure 3 and Table 1). In contrast, 2-ClE_2_ and 4-ClE_2_ (2.5 mg and 5.0 mg) did not induce mammary tumors even after one year of treatment. The 5.0 mg of chlorinated estrogens was 4-times higher dose than 1.25 mg E_2_ that induced mammary tumors [27], indicating that the chlorinated estrogens appear to be less carcinogenic. The 17α-ethinyl form of E_2_ (EE_2_) was reported by another research group [28,34], to be a strong inducer of mammary tumors in ACI rats. However, both 2-ClEE_2_ and 4-ClEE_2_ did not develop mammary tumors (Table 1). The less carcinogenic potential observed for chlorinated E_2_ or EE_2_ was supported by histological examination of mammary glands of chlorinated estrogen-treated ACI rats (Figure 4).

In our recent study [27], 2-fluoro-17β-estradiol (2-FE_2_) did not induce mammary tumors whereas 4-fluoro-17β-estradiol (4-FE_2_) did. Because of the carbon–fluorine bond strength [35], fluorination inhibits metabolic hydroxylation of E_2_. A fluorine at the 2-position of E_2_ prevents 2-hydroxylation, resulting in preferential 4-hydroxylation [36]. On the other hand, a fluorine at the 4-position of E_2_ prevents 4-hydroxylation, resulting in preferential 2-hydroxylation. These results suggest that the development of mammary tumors might occur through the 2-hydroxylation of 4-FE_2_, not through the 4-hydroxylation of 2-FE_2_. Surprisingly, unlike 4-FE_2_, 4-ClE_2_ did not induce mammary tumors. Because a chlorine atom has greater steric hindrance and electron-withdrawing power than a fluorine atom, chlorine modification of estrogen may be more effective to diminish its carcinogenicity. The bulky chlorine atom may affect to inhibit the hydroxylation at both the 2- and 4-positions of estrogens. In addition, the resulting catecholestrogens may hardly be oxidized by the bulky chlorine atom to form their reactive quinones that can damage DNA [19,21,23].

To evaluate such evidence, the metabolites of halogenated estrogen absorbed into body should be determined. Using a radioimmunoassay (RIA), the serum E_2_ level in ACI rats treated with 3.0 mg E_2_ pellet was reported to be less than 175 pg/mL [25]. Unfortunately, the RIA used for E_2_ is not applicable to assay other estrogens. A newly sensitive method is required to be developed for determining such low level of halogenated estrogens and their metabolites, especially in mammary and reproductive organs.

To determine whether the chlorinated estrogens have estrogenic potency, the uterotrophic activity was measured as an indicator after treating OVX-rats subcutaneously for 3 days with 2-ClE_2_ or 4-ClE_2_. Significant uterotrophic activity was observed when treated with 34 μg of either 2-ClE_2_ or 4-ClE_2_ (Figure 5A). The body weight of OVX-rats was ~180 g; therefore, the daily dose 34 μg/rat was 189 μg/kg/day. Because the body weight of ACI rats treated for 52 weeks with a 5.0 mg chlorinated estrogen was ~80 g, the daily dose was estimated to be 172 μg/kg/day that was similar to that (189 μg/kg/day) of OVX-rats treated with 34 μg chlorinated estrogens. This indicated that the dose appearing high estrogenic potential may not always have carcinogenic potency.

E_2_ is generally given by parenteral treatment because if taken by mouth it is inactivated quickly. On the other hand, the 17α-ethinyl form can be absorbed more efficiently by the body and thus has a higher bioavailability after oral delivery [29,30]. In OVX-rats treated orally with either 2-ClEE_2_ or 4-ClEE_2_, both chlorinated compounds showed significant uterotrophic activity (Figure 5B). Although the chlorinated estrogens required higher doses than EE_2_, they provided effective estrogenic potency by oral treatment.

In our previous paper [27], both non-carcinogenic 2-FE_2_ and carcinogenic 4-FE_2_ appeared similar uterotrophic potency, indicating that estrogenic potential may not be the sole factor driving mammary tumorigenesis. 2-FE_2_ retaining estrogenic potential has shown to be a safer alternative for HRT. However, the difficult isolation process of 2-FE_2_ from 4-FE_2_ [37] requires the development of a specific separation method. On the other hand, both 2-ClE_2_ and 4-ClE_2_ having estrogenic potency did not present carcinogenic potency. If no separation method is established, the mixture of chlorinated estrogens may be used similarly to a combination tablet currently prescribed for HRT.

In conclusion, chlorinated estradiol derivatives retaining estrogenic potential were less mammary carcinogenic. Such chlorinated estrogens could be used as safer alternatives to carcinogenic estrogens now in use for HRT.

## 4. Materials and Methods

### 4.1. Chemicals

E_2_ [estra-1,3,5(10)-triene-3,17β-diol], EE_2_ [17α-ethinylestra-1,3,5(10)-triene-3,17β-diol] and cholesterol were purchased from Fujifilm Wako Pure Chemical Corp. (Osaka, Japan). Trichloroisocyanuric acid (TCCA) was purchased from Tokyo Chemical Industry Co., Ltd. (Tokyo, Japan).

### 4.2. Synthesis of Chlorinated Estrogens

2-ClE_2_, 4-ClE_2_, 2-ClEE_2_, and 4-ClEE_2_ were synthesized in good yield by a following modification of the established procedure [38]. TCCA (0.84 g) was added to an ice-cooled solution of the E_2_ (2.0 g) in acetonitrile (100 mL) and stirred for 1 hr. The reaction mixture was extracted with ethyl acetate. The organic layer was washed with 5% aqueous sodium hydrogen sulphite, 5% aqueous sodium carbonate and water, and evaporated. An ethanol solution (40 mL) of the residue was treated with sodium borohydride (0.67 g), and stirred at room temperature for 20 min. The reaction mixture was extracted with ethyl acetate. The organic layer was evaporated to give a residue, which was submitted to preparative HPLC equipped with an ODS column to given pure products of 2-ClE_2_ (0.22 g, 10%), 4-ClE_2_ (0.26 g, 11%), and 2,4-dichloro-17β-estradiol (2,4-diClE_2_; 0.22 g, 9%). Using the same procedure as described for chlorination of E_2_, treatment of EE_2_ (2.0 g) with TCCA (0.80 g) gave 2-ClEE_2_ (0.21 g, 9%), 4-ClEE_2_ (0.26 g, 12%), and 2,4-diClEE_2_ (0.30 g, 12%). Preparative HPLC conditions were as follows. Pump, model SP-22 (Tokyo Rikakikai, Co., LTD., Tokyo, Japan); detector, model 8011 (Tosoh Corp., Tokyo, Japan) at 270 nm; column, two 22 mm i.d. × 300 mm glass columns containing μ-Bondasphere 15 μm (Waters, Milford, MA, USA) were joined together; mobile phase, methanol—water (80:20, v/v); flow rate, 5.0 mL/min. By HPLC/UV or NMR analysis, the purity of chlorinated estrogens was determined to be >99%.

### 4.3. Tumorigenesis of Chlorinated Estrogens

The animal studies were approved by an ethics committee of Faculty of Pharmacy, Meijo University. All procedures with animals were conducted in compliance with the Guidelines for Proper Conduct of Animal Experiments (Science Council of Japan). Rats (ACI, 5-week-old females, Harlan) were given water and food ad libitum and kept on a 12-h light/dark cycle throughout the study. Following an established protocol for E_2_ [25,27,31], after one week of acclimation, the following pellet was implanted under the dorsal skin with light isoflurane anesthesia: 2-ClE_2_ 2.5 mg or 5.0 mg (*n* = 6 rats), 4-ClE_2_ 2.5 mg or 5.0 mg (*n* = 6), 2-ClEE_2_ 2.5 mg or 5.0 mg (*n* = 5–6), or 4-ClEE_2_ 2.5 mg or 5.0 mg (*n* = 5) in cholesterol (15.0 or 17.5 mg), and 20 mg cholesterol alone (*n* = 6) as the negative control. ACI rats implanted with E_2_ 1.25 mg (*n* = 5), 2.5 mg (*n* = 10), or 5.0 mg (*n* = 5) pellet were used as the positive control. Development of mammary tumors was monitored by palpation once a week for 52 weeks. At the end of experiments, rats were euthanized by CO_2_ asphyxiation. Pathological determination of mammary tumors was performed following the established procedure in our laboratory [27,31].

### 4.4. Mammary Whole-Mount Preparation and Morphometric Analysis

Rats were euthanized under isoflurane anesthesia. Following an established protocol [27,31], the skin containing mammary glands was collected and fixed in 10% neutral-buffered formalin for at least 3 days. The mammary glands were then dissected free from the skin and processed as a whole mount. The glands were defatted in ethanol, acetone, chloroform, and ethanol again for at least 3 days in each solvent. After rehydration, the glands were stained with hematoxylin and washed with distilled water. The stained glands were cleaned up manually by viewing through a stereomicroscope, dehydrated in ethanol, cleared in xylene and mounted. Photographs were taken using a digital camera (Olympus, Tokyo, Japan) mounted on a stereomicroscope (Stemi SV11, Carl Zeiss, Jena, Germany).

### 4.5. Determination of Uterotrophic Potential

The uterotrophic activity of chlorinated estrogens was determined by following the protocol reported previously [27,31]. Briefly, OVX-rats (Sprague-Dawley, 6-week-old females, Japan SLC, Inc., Shizuoka, Japan; 4 rats/dose) were treated subcutaneously for 3 days with 2-ClE_2_ or 4-ClE_2_. A dose one- or ten-times molar equivalent to E_2_ (3.0 μg/rat/day) was used; 3.4 or 34 μg/rat/day for 2-ClE_2_ or 4-ClE_2_. The ethinyl compounds 2-ClEE_2_ and 4-ClEE_2_ were administered orally. A dose one- or three-times molar equivalent to EE_2_ (16.5 μg/rat/day) was used; 18 or 54 μg/rat/day for 2-ClEE_2_ or 4-ClEE_2_. The negative control rats received vehicle only. On day 4, uterine horns were dissected and trimmed of fascia and fat. The uterine weight was measured after removing the luminal fluids on filter paper. Uterine wet-weight to body-weight (bw) ratios (mg/g bw) were compared with that obtained for the OVX-rats treated subcutaneously with E_2_ (3.0 μg/rat/day) as a positive control. Statistical analysis (one-way ANOVA with Tukey’s post hoc test) was performed to evaluate the significance of the differences in treatment effects.

## Figures and Tables

**Table 1:** Incidence of mammary tumors in ACI rats implanted with a chlorinated estrogen.

Compound	Dose (mg/pellet)	Cumulative Incidence (No. of Rats) ^a^	Percentage of Rats with Tumors (%)
Control	-	0/6	0
E_2_	1.25	4/5	80
	2.5	9/10	90
	5.0	- ^b^	- ^b^
2-ClE_2_	2.5	0/6	0
	5.0	0/6	0
4-ClE_2_	2.5	0/6	0
	5.0	0/6	0
2-ClEE_2_	2.5	0/5	0
	5.0	0/6	0
4-ClEE_2_	2.5	0/5	0
	5.0	0/5	0

^a^ Data are expressed as number of tumor-induced rats per total number of rats used. ^b^ Due to the severe loss of body weight, the experiment was terminated.

## Data Availability

Not applicable.

## Abbreviations

E_2_	17β-estradiol
2-OHE	2-hydroxyestrogen
4-OHE	4-hydroxyestrogen
2-ClE_2_	2-chloro-17β-estradiol
4-ClE_2_	4-chloro-17β-estradiol
EE_2_	17α-ethinylestradiol
2-ClEE_2_	2-chloro-17α-ethinylestradiol
4-ClEE_2_	4-chloro-17α-ethinylestradiol
2-FE_2_	2-fluoro-17β-estradiol
4-FE_2_	4-fluoro-17β-estradiol
ACI	August Copenhagen Irish
HRT	Hormone replacement therapy
OVX	Ovariectomized
bw	Body weight
RIA	Radioimmunoassay
